# Improvement of the Thermal Performance of the GaN-on-Si Microwave High-Electron-Mobility Transistors by Introducing a GaN-on-Insulator Structure

**DOI:** 10.3390/mi15121525

**Published:** 2024-12-21

**Authors:** Lu Hao, Zhihong Liu, Hanghai Du, Shenglei Zhao, Han Wang, Jincheng Zhang, Yue Hao

**Affiliations:** 1 School of Microelectronics, Xidian University, Xi’an 710071, China; haolu0614@outlook.com (L.H.); slzhao@xidian.edu.cn (S.Z.); jchzhang@xidian.edu.cn (J.Z.); yhao@xidian.edu.cn (Y.H.); 2 Guangzhou Institute of Technology, Xidian University, Guangzhou 510555, China; 3 Department of Electrical and Electronic Engineering, University of Hong Kong, Hong Kong; hanwang6@hku.hk

**Keywords:** GaN-on-Si, GNOI, HEMT, thermal performance, bonding dielectric

## Abstract

GaN-on-Si high-electron-mobility transistors have emerged as the next generation of high-powered and cost-effective microwave devices; however, the limited thermal conductivity of the Si substrate prevents the realization of their potential. In this paper, a GaN-on-insulator (GNOI) structure is proposed to enhance the heat dissipation ability of a GaN-on-Si HEMT. Electrothermal simulation was carried out to analyze the thermal performance of the GNOI-on-Si HEMTs with different insulator dielectrics, including SiO_2_, SiC, AlN, and diamond. The thermal resistance of the HEMTs was found to be able to be obviously reduced and the DC performance of the device can be obviously improved by removing the low-thermal-conductivity III-nitride transition layer and forming a GNOI-on-Si structure with SiC, AlN, or diamond as the bonding insulator dielectrics.

## 1. Introduction

GaN-based high-electron-mobility transistors (HEMTs) are highly attractive for RF applications due to GaN-based materials’ unique properties such as high critical electric fields, high polarization-coefficient-induced two-dimensional electron gas (2DEG) density, high electron saturation velocity, etc. [1,2,3,4,5,6,7,8]. GaN epitaxial materials grown on a Si substrate have the advantages of low substrate cost, large wafer scale, and the capability of being fabricated in a mature Si CMOS foundry, so GaN-on-Si HEMTs have emerged as one of the most powerful candidates to be implemented in the next generation of radar, communication base stations, and mobile ends [5,6,7,8]. An excellent performance of a cut-off frequency (*f*_T_) of 310 GHz [1] and an output power density of 12.88 W/mm at 2.14 GHz [2], 7 W/mm at 10 GHz [3], and 2 W/mm at 40 GHz [4] have been reported for GaN-on-Si HEMTs. However, compared to the reported RF performance of GaN HEMTs on SiC substrates, such as the *f*_T_ of 454 GHz [5], 40 W/mm at 4 GHz [6], 16.5 W/mm at 10 GHz [7], and 10.5 W/mm at 40 GHz [8], the potential of the GaN-on-Si HEMTs has not been fully realized.

One of the main hurdles limiting the RF performance of a GaN-on-Si HEMT is its relatively poorer heat dissipation ability and higher thermal resistance compared to GaN-on-SiC. There are mainly two physical mechanisms responsible for the poor thermal performance of GaN-on-Si microwave transistors. First, the typical thermal conductivity of a SiC substrate at room temperature is up to 4.0 W/cm·K, while that of a Si substrate is only around 1.5 W/cm·K [9]. Second, due to the large lattice mismatch between GaN and Si, thick transition layers with poor thermal conductivity are needed between the GaN buffer and the Si substrate [1,2,3,4,10].

To date, a few technical routes have been reported to improve the heat dissipation performance of the GaN-on-Si microwave transistors. One of them is to deposit a high-thermal-conductivity layer like AlN, BN, and nanocrystalline diamond on the front surface of the devices [11,12,13]. In addition, thinning the Si substrate of the fabricated device and then transferring the device onto a heat sink with high thermal conductivity has been proposed [14]. The third approach is to optimize the layout design, including increasing the gate finger pitch and reducing the hot spot density [15]. These approaches can enhance the thermal performance of the GaN-on-Si RF devices to some degree, but there is still a lack of satisfactory measures to address the problem.

In this work, we propose a GaN-on-insulator (GNOI) structure to enhance the heat dissipation ability of GaN-on-Si HEMTs. In conventional GaN-on-Si HEMTs, the GaN epilayers include thick transition layers, which could be a few layers of AlGaN with varied Al composition, or a GaN/AlN super-lattice. The transition layers have a poor thermal conductivity due to the large density of dislocations [10]. In the GNOI structure proposed here, the transition layers were removed and the GaN barrier, channel, and buffer layers were transferred onto another Si substrate through wafer bonding technology using a high-thermal-conductivity dielectric bonding layer.

The thermal performance of the GNOI-on-Si HEMTs will be analyzed through TCAD electrothermal simulation. The advantages of this approach include the full process compatibility with large wafer mass production foundries, and also the possibility to realize monolithic integration of Si CMOS digital circuits [16].

## 2. Electrothermal Simulation Models

**Figure 1:**
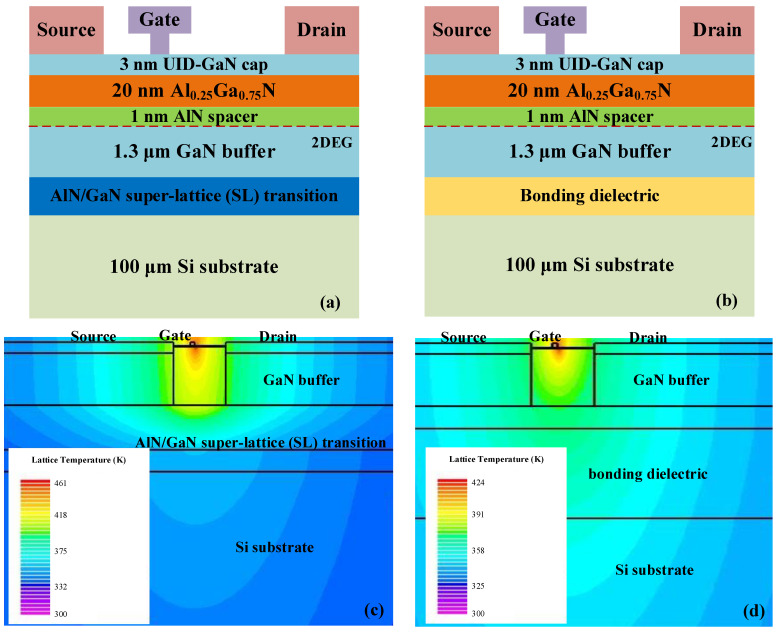
The schematics of the cross sections of the (**a**) conventional GaN-on-Si and (**b**) proposed GNOI-on-Si RF HEMT. Simulated lattice temperature distribution of (**c**) a conventional GaN-on-Si HEMT and (**d**) a GaN-on-Insulator (GNOI) HEMT with 2 μm AlN as the bonding dielectric at *V*_G_ = 0 V and *V*_DS_ = 10 V.

The schematic of the cross section of a conventional GaN-on-Si microwave transistor used in the electrothermal simulation is shown in Figure 1a. The GaN epitaxial layers include an AlN nucleation layer, an AlN/GaN super-lattice (SL) transition layer, a GaN buffer, a thin AlN spacer, an AlGaN or InAlN barrier, and a GaN cap, following the structure reported in [17]. The GaN epilayers are grown on a high-resistivity Si (111) substrate. Figure 1b shows the cross-sectional schematic of the proposed GNOI-on-Si HEMT structure. As the transition layer composed of AlN/GaN super-lattices (SLs) has a much lower thermal conductivity than the thick GaN material [10], the III-nitride transition layer is removed and the remaining nitride epilayers are transferred onto another Si substrate through the wafer bonding technology. The bonding dielectric materials can be SiO_2_, SiC, AlN, diamond, etc. The fabrication flow of such a GNOI-on-Si structure has been reported elsewhere [16].

The GaN RF HEMT in the electrothermal simulation had a gate length *L*_G_ of 0.25 μm, a source-drain distance *L*_SD_ of 3.0 μm, and a gate width *W*_G_ of 50 μm. The gate Schottky barrier height was set to 5.1 eV. The Si substrate was thinned down to 100 μm.

Three-dimensional electrothermal simulations were carried out using the device simulator ATLAS from Silvaco TCAD 2019, Santa Clara, CA, USA. The bottom of the Si substrate was set to a fixed temperature of 300 K. The thermal conductivities of the various regions in the device are considered to be temperature-dependent with a model of

(1) $${k(T)=k}_{\mathrm{RT}}{\left(300/(273+T)\right)}^{\alpha },$$

where *k*(*T*) and *k_RT_* are the thermal conductivity values at the temperature *T* and room temperature, respectively; *α* is the power index. Table 1 lists the thermal conductivity values of the bonding dielectrics at 300 K with the various thicknesses used in the simulation [17,18,19,20,21]. Other material parameters and physics models used in the simulation were based on those reported in reference [9], where the simulation had been validated through comparisons with experimental device performance.

In addition, the thermal conductivity values of the AlGaN barrier, GaN buffer, transition layer, and Si substrate are set to 0.3 W/cm·K, 1.6 × (300/(273 + *T*))^1.4^ W/cm·K, 0.1 W/cm·K, and 1.48 × (300/(273 + *T*))^1.65^ W/cm·K, respectively [9,10,22,23]. Si_3_N_4_ with a thermal conductivity of 0.2 W/cm·K is considered for device surface passivation. The thermal boundary conductivity between GaN materials and dielectrics or Si was simulated following the method in [9]. The polarization coefficient of the barrier materials and the channel mobility were adjusted to make the simulated DC output characteristics at low drain biases match with the experimental data [18].

## 3. Simulation Results and Discussion

It is widely recognized that it is essential to select a material with thermal conductivity for the layers between active regions and substrates to enhance the devices’ heat dissipation ability. Typically, these high-thermal-conductivity layers are deposited through epitaxial growth, which is often constrained by lattice structure compatibility. There are limited reports available on the use of high-thermal-conductivity dielectric material as insertions between active regions and substrates, e.g., using Al_2_O_3_ bonding material for GaSb-based lasers on an SOI wafer [24]. In this work, we propose a novel GaN-on-insulator (GNOI) structure. The transition layers with low thermal conductivity were removed and the GaN barrier, channel, and buffer layers were transferred onto another Si substrate through wafer bonding technology using a high-thermal-conductivity dielectric bonding layer.

Figure 1c,d show the simulated lattice temperature distribution in a conventional GaN-on-Si HEMT and a GNOI-on-Si HEMT with 2 μm AlN as the bonding dielectric at gate voltage *V*_G_ = 0 V and drain voltage *V*_DS_ = 10 V. The hot spots with temperatures of 188 °C and 151 °C are located at the AlGaN/GaN interface at the gate edge close to the drain. This agrees with the fact that the peak electric field formed due to a depletion region formed in the 2DEG channel at the gate edge close to the drain.

**Figure 2:**
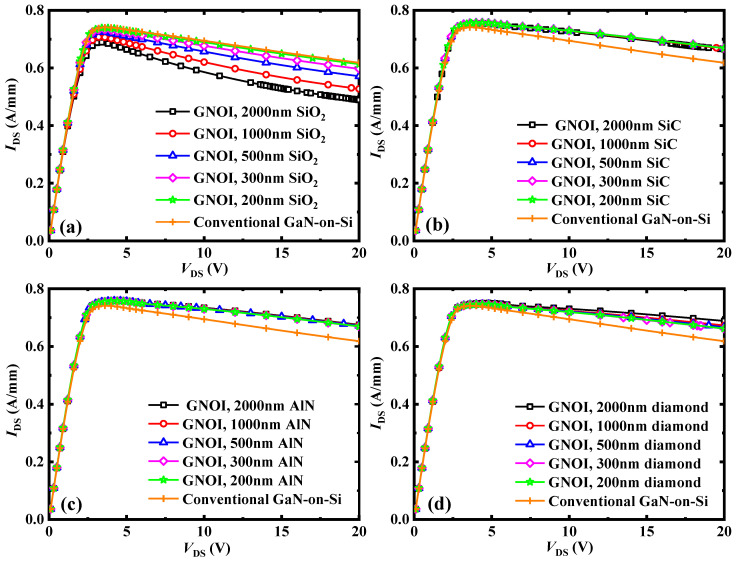
Simulated DC output characteristics of the conventional GaN-on-Si HEMT and GNOI-on-Si HEMTs with various bonding dielectrics and a thickness of *V*_G_ = 0 V considering the self-heating effect. (**a**) SiO_2_; (**b**) SiC; (**c**) AlN; (**d**) diamond.

Figure 2 shows the simulated DC output characteristics of the conventional GaN-on-Si HEMT and the GNOI-on-Si HEMTs with various bonding dielectrics at different thickness at *V*_G_ = 0 V considering the self-heating effect. Influenced by the self-heating effect, as the drain voltage increases the DC output currents of the devices show a downward trend in the saturation region as well as negative resistances. The current drop increases significantly with the bonding dielectric thickness for GNOI-on-Si HEMTs using SiO_2_ as the bonding dielectric, whereas the thickness has a negligible effect on the current drop for SiC, AlN, and diamond. After considering the fabrication process to address challenges such as wafer bow, surface roughness, residues, etc., a bonding dielectric thickness of 2 μm was selected for investigating the impact of various bonding dielectrics on the device’s DC characteristics; however, the trend is also applicable to other thicknesses. In the conventional GaN-on-Si and GNOI-on-Si HEMTs with 2 μm SiO_2_, SiC, AlN, and diamond bonding dielectrics, the values of the current drop are 17%, 29%, 12%, 12%, and 8%, respectively. The GNOI-on-Si HEMT with a diamond bonding dielectric shows the best DC performance.

**Figure 3:**
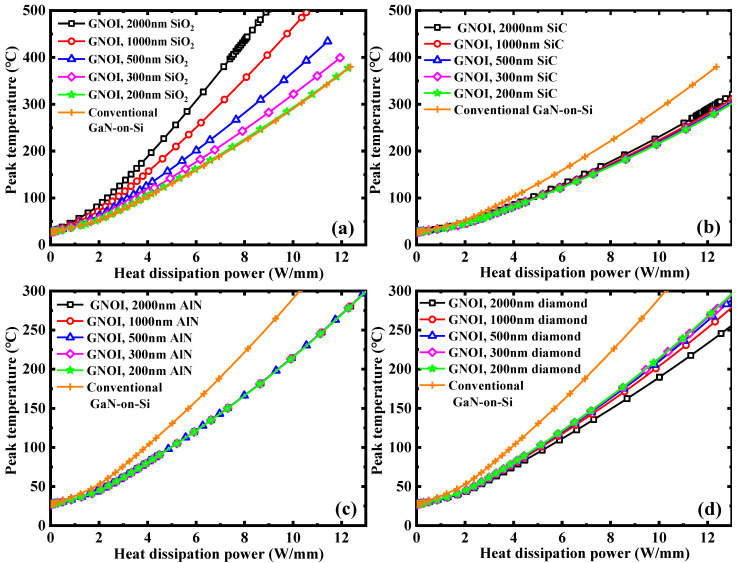
Simulated peak temperature as a function of heat dissipation power in GNOI-on-Si HEMTs with various bonding dielectrics and thickness. (**a**) SiO_2_; (**b**) SiC; (**c**) AlN; (**d**) diamond.

Figure 3 shows the simulated peak temperature as a function of heat dissipation power in GNOI-on-Si HEMTs with various bonding dielectrics and thicknesses. With the increase of the thickness of the bonding dielectrics from 200 nm to 2 μm, the heat dissipation of the GNOI-on-Si HEMT becomes better in the case of diamond, while similar for SiC and AlN, and worse for SiO_2_. The reason for the worse heat dissipation from the GNOI-on-Si HEMT with the thicker SiO_2_ bonding dielectric is due to the much smaller thermal conductivity of thin SiO_2_ material compared to that of the Si substrate. It is worth noting that although the thermal conductivity value of the single-crystal SiC is as high as 4.0 W/cm·K [10], the deposited SiC thin film using a conventional PECVD tool or a sputter tool is generally amorphous and has a much lower thermal conductivity than that of the single-crystal SiC [19]. The heat dissipation ability of the GNOI-on-Si HEMT with a SiC bonding dielectric does not show improvement with the increase of the SiC layer thickness. In a similar way, there is almost no change in the heat dissipation ability of the GNOI-on-Si HEMT with a thick AlN bonding layer. However, with regard to a thin layer of diamond, even though the thermal conductivity value of a poly-crystalline or nano-crystalline diamond is much lower than the single-crystal, it is still obviously higher than the Si substrate [22], so a thick diamond bonding layer will improve the heat dissipation in a GNOI-on-Si HEMT.

**Figure 4:**
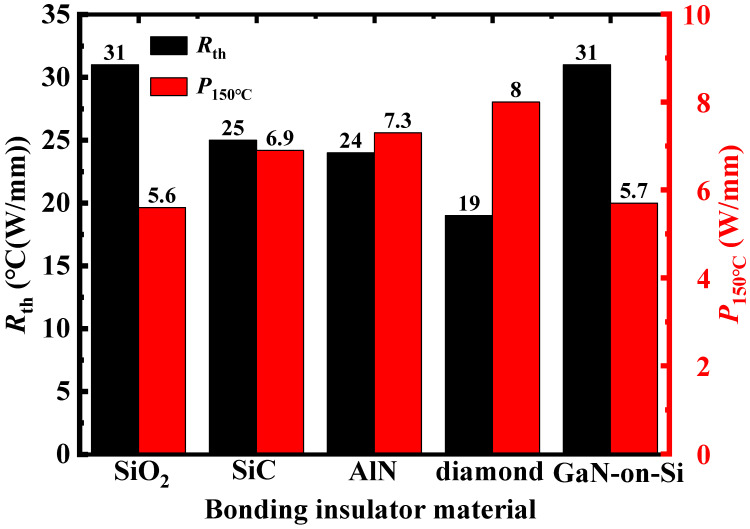
Simulated thermal resistance *R*_th_ and *P*_150 °C_ in the conventional GaN-on-Si HEMT and GNOI-on-Si HEMTs with various bonding dielectrics (SiO_2_: 200 nm; SiC: 2 μm; AlN: 2 μm; diamond: 2 μm).

The thermal resistance of a semiconductor device is defined as:

(2) $$R_{\mathrm{th}\;}=\frac{T_{j}-T_{R}}{P_{\mathrm{dis}}}$$

where *T*_j_ is the junction temperature when the device temperature reaches steady state, *T_R_* is the reference point temperature and assumed to be room temperature, and *P*_dis_ is the heat dissipation power. *R*_th_ values of the GaN-on-Si and GNOI-on-Si HEMTs were extracted and illustrated in Figure 4. *R*_th_ of 31, 31, 25, 24, and 19 °C (W/mm) were found for the conventional and GNOI devices with 200 nm SiO_2_, 2 μm SiC, 2 μm AlN, and 2 μm diamond bonding dielectrics, respectively. The *R*_th_ of the GNOI devices with 2 μm AlN and diamond bonding layers are 23%, 40% lower than that of the conventional device.

The maximum heat dissipated power density allowable at a given temperature limit is a common concern for a device in real applications. The maximum power density *P*_150 °C_ at a peak temperature of 150 °C is summarized in Figure 4. The values of *P*_150 °C_ were 5.7, 5.6, 6.9, 7.3, and 8 W/mm for the conventional and GNOI devices with 200 nm SiO_2_, 2 μm SiC, AlN, and diamond bonding dielectrics, respectively. Compared to the conventional GaN-on-Si HEMTs, the proposed GNOI-on-Si HEMTs with SiC, AlN, and diamond bonding dielectrics can effectively increase the device’s thermal performance.

It is worth noting that semiconductor wafer bonding through various dielectrics, such as SiO_2_, SiC and AlN, has been successfully demonstrated in experiments [16,25,26]. Wafer bonding through diamond remains a significant challenge but it is now an active area of research [27,28]. The proposed technique in this work is considered feasible.

**Figure 5:**
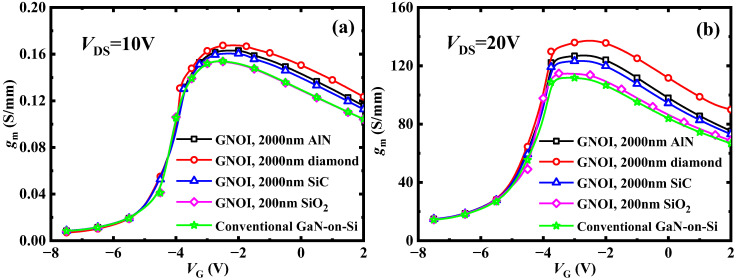
Electrothermally simulated DC transfer characteristics at (**a**) *V*_DS_ = 10 V and (**b**) *V*_DS_ = 20 V.

Figure 5 shows the electrothermal simulation of DC transfer characteristics at (a) *V*_DS_ = 10 V and (b) *V*_DS_ = 20 V for the conventional GaN-on-Si and proposed GNOI-on-Si HEMTs with various bonding dielectrics. The transconductance, *g*_m_, is strongly associated to the RF performance parameters such as power gain, etc. Compared with the conventional GaN-on-Si HEMT, the *g*_m_ of the proposed GNOI-on-Si HEMTs with SiC, AlN, or diamond bonding dielectrics can be obviously improved, especially at a higher drain bias. As Figure 5b shows, the values of the peak transconductance *g*_mmax_ are improved by 2%, 10%, 13%, and 22% for the GNOI devices with 200 nm SiO_2_, 2 μm SiC, AlN, and diamond bonding dielectrics, respectively, compared to the conventional GaN-on-Si.

## 4. Conclusions

In conclusion, a GNOI-on-Si structure is proposed to improve the heat dissipation in conventional GaN-on-Si microwave devices. The thermal performance of the GNOI-on-Si HEMT with various bonding dielectrics including SiO_2_, SiC, AlN, and diamond was studied through electrothermal simulation. By removing the low-thermal-conductivity nitride transition layer in the GaN-on-Si HEMT structure and introducing a high-thermal-conductivity bonding electric layer such as SiC, AlN, or diamond, the heat dissipation ability of the GNOI-on-Si HEMT can be obviously improved, including a lower current drop at high drain bias, a lower thermal resistance, a higher power handling index at a fixed channel temperature, a higher transconductance, etc. These results show the great potential of GNOI-on-Si HEMTs in high-power microwave applications.

## Figures and Tables

**Table 1:** Thermal conductivity values of the bonding dielectrics at 300 K used in the simulation.

Material	Thermal Conductivity (W/cm·K)	Thickness (μm)
SiO_2_	0.012/0.012/0.012/0.012/0.012	0.2/0.3/0.5/1.0/2.0
SiC	0.014/0.64/0.64/0.64/0.64	0.2/0.3/0.5/1.0/2.0
AlN	1.0/1.0/1.05/1.2/1.3	0.2/0.3/0.5/1.0/2.0
Diamond	2.12/2.18/2.3/2.6/3.7	0.2/0.3/0.5/1.0/2.0

## Data Availability

The original contributions presented in this study are included in the article. Further inquiries can be directed to the corresponding author.
